# Can Musical Training Influence Brain Connectivity? Evidence from Diffusion Tensor MRI

**DOI:** 10.3390/brainsci4020405

**Published:** 2014-06-10

**Authors:** Emma Moore, Rebecca S. Schaefer, Mark E. Bastin, Neil Roberts, Katie Overy

**Affiliations:** 1 Institute of Music in Human and Social Development (IMHSD), Reid School of Music, Alison House, 12 Nicolson Square, Edinburgh EH8 9DF, UK; E-Mail: e.v.moore@sms.ed.ac.uk; 2 SAGE Center for the Study of the Mind, University of California, Santa Barbara, CA 93106, USA; E-Mail: rebecca.schaefer@sagecenter.ucsb.edu; 3 Centre for Clinical Brain Science, University of Edinburgh, Edinburgh EH8 9YL, UK; E-Mail: mark.bastin@ed.ac.uk; 4 Clinical Research Imaging Centre (CRIC), University of Edinburgh, Edinburgh EH8 9YL, UK; E-Mail: neil.roberts@ed.ac.uk

**Keywords:** musicians, white matter, diffusion tensor MRI, neuroplasticity

## Abstract

In recent years, musicians have been increasingly recruited to investigate grey and white matter neuroplasticity induced by skill acquisition. The development of Diffusion Tensor Magnetic Resonance Imaging (DT-MRI) has allowed more detailed investigation of white matter connections within the brain, addressing questions about the effect of musical training on connectivity between specific brain regions. Here, current DT-MRI analysis techniques are discussed and the available evidence from DT-MRI studies into differences in white matter architecture between musicians and non-musicians is reviewed. Collectively, the existing literature tends to support the hypothesis that musical training can induce changes in cross-hemispheric connections, with significant differences frequently reported in various regions of the corpus callosum of musicians compared with non-musicians. However, differences found in intra-hemispheric fibres have not always been replicated, while findings regarding the internal capsule and corticospinal tracts appear to be contradictory. There is also recent evidence to suggest that variances in white matter structure in non-musicians may correlate with their ability to learn musical skills, offering an alternative explanation for the structural differences observed between musicians and non-musicians. Considering the inconsistencies in the current literature, possible reasons for conflicting results are offered, along with suggestions for future research in this area.

## 1. Introduction

Playing a musical instrument requires a host of specialised skills, including translating written notation into motor movements, precise timing, bimanual coordination and rapid auditory processing skills. These specialist skills are developed through a considerable volume of practice and training, which normally commences in early childhood. The large body of training musicians undergo has led to the suggestion that these individuals are an ideal group to investigate training-induced structural brain plasticity as a consequence of learning a new skill [1,2]. Over the last 20 years, both structural and functional differences between brains of musicians and non-musicians, as well as between different types of musicians, e.g., violinists and pianists [3], have been reported. Many of the early studies into musical training-induced neuroplasticity used segmentation to investigate grey matter (GM) differences on structural MRI, with increased GM volume reported in musicians compared with non-musicians e.g., in primary motor cortex, premotor cortex [4], Heschl’s gyrus [5] and cerebellum [6]. For reviews see Tervaniemi (2009) [7], Jäncke (2009) [8], Wan and Schlaug (2010) [9] and Herholz and Zatorre (2012) [10]. More recently, an increasing amount of research has focused on white matter (WM) differences between musicians and non-musicians; the underlying biological mechanisms involved in differences in WM structure may include increases in volume, organisation, degree of myelination and functional connectivity of tracts linking together different cortical regions. Much research to date in this area has focused particularly on cross-hemispheric connections, *i.e.*, corpus callosum, but other tracts of interest have included intra-hemispheric (association) connections such as the arcuate fasciculus (AF; temporal/parietal to frontal cortex), superior longitudinal fasciculus (SLF; temporal/parietal to frontal cortex), inferior longitudinal fasciculus (ILF; temporal to occipital cortex) and uncinate fasciculus (UF; hippocampus and amygdala to frontal cortex), and fibres related to motor function such as the corticospinal tracts (CST) and cerebellar peduncles. The varying results found by the studies published to date currently make it difficult to draw clear conclusions about the effects of musical training, not least because the analysis methods employed and the type and number of musicians recruited varies considerably across studies, often limiting the extent to which results can be directly compared. The majority of research into WM architecture in musicians has been published within the last five years and has not yet been reviewed in detail. Here, the findings on structural brain plasticity and musical training presented to date will be discussed, focusing specifically on measures of WM connectivity obtained from Diffusion Tensor Magnetic Resonance Imaging (DT-MRI), and with the aim of highlighting any common trends that can be identified.

### Investigation of White Matter Using Structural MRI

Structural MRI data were used for the initial investigations into WM differences between musicians and non-musicians. Schlaug and colleagues [11] first reported that when divided into seven segments, the anterior half (segments 1–4) of the corpus callosum (CC), a dense bundle of WM fibres responsible for inter-hemispheric communication and connecting brain areas including the premotor, supplementary motor and motor cortices, was significantly larger in professional musicians (*n* = 30) compared with non-musicians (*n* = 30). Moreover, the anterior half of the CC was significantly larger in a subgroup of musicians who commenced their training before the age of seven, compared with both the musicians who started training after the age of seven and non-musicians. This finding is often cited as evidence for a sensitive period of development for musical training during which the brain has the greatest potential to undergo neuroplastic change, and has been supported by additional cross-sectional studies comparing early trained (ET) with late trained (LT) musicians [12]. A subsequent longitudinal study from Schlaug and colleagues [13,14] found no pre-existing differences in CC size in 5–7 year old children about to begin musical training (*n* = 50) compared with a matched group of children not intending to take music lessons (*n* = 25), and no differences in the first group of children re-scanned after an average of 14 months [13]. However, after approximately 29 months of instrumental training, an ROI analysis of segments 3–6 of the CC found that segment 3 was significantly larger in the remaining high practising children (*n* = 6), compared with both the low practising children (*n* = 12), and the children who did not receive any instrumental training (*n* = 13) [14]. Meanwhile, an analysis conducted by Hyde and colleagues [15] used Deformation-Based Morphometry (DBM) across the whole brain to examine a matched sample of these participants at the earlier scanning time point and found that after an average of 15 months of musical training, children in the instrumental group (*n* = 15) showed a greater relative voxel size in the midbody (segments 4 and 5) of the CC, compared with children who did not receive instrumental training (*n* = 16). Although the precise region of the CC found to show significant differences was slightly different with each type of analysis and at different time points, the findings from this longitudinal study provide evidence of structural WM changes occurring in correlation with musical training in children. In addition, collectively these studies indicate that WM neuroplasticity in the CC may be related to both age of training onset and amount of practice.

## 2. Diffusion Tensor MRI

Recently, the development of DT-MRI has allowed further exploration of whether musical training can induce WM changes in the brain, using measures of WM diffusivity rather than WM volume. Thus far, there are relatively few DT-MRI studies specifically investigating WM differences in musicians and whilst there is some evidence that musicians exhibit WM differences compared with non-musicians, there are also a number of contradictory findings. Firstly DT-MRI techniques, including those aspects that are relevant to a review of the literature will be briefly discussed. Next, the reported findings will be considered, followed by a more general discussion of the discrepancies, and implications for future work.

### 2.1. Overview

**Figure 1:**
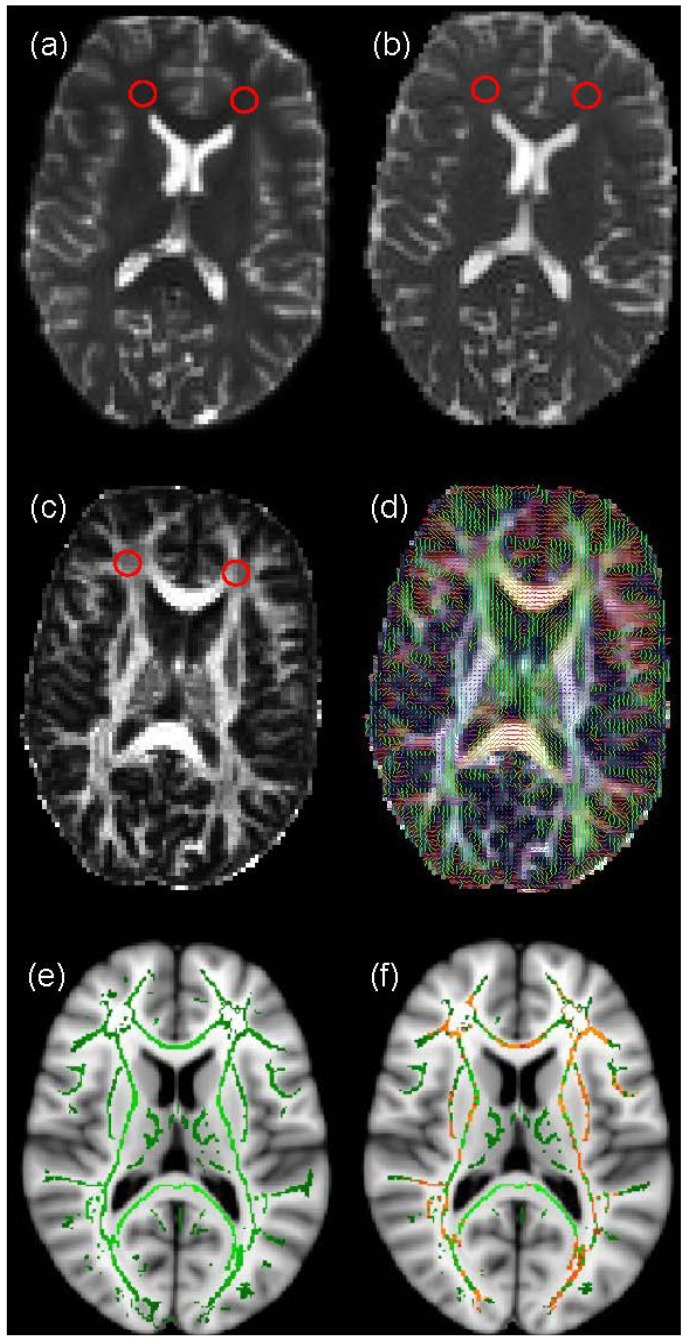
An example of Diffusion Tensor Magnetic Resonance Imaging (DT-MRI) analysis. The figure displays maps of (**a**) T_2_-weighted signal intensity, (**b**) mean diffusivity (MD), (**c**) fractional anisotropy (FA) and (**d**) colour-coded principal diffusion direction overlaid on FA from a normal volunteer obtained using FMRIB’s Diffusion Toolbox (FDT) analysis pipeline [24]. Region of Interest (ROIs) placed in frontal white matter (WM) in (**a**) and transferred to (**b**) and (**c**) for measurement of MD and FA are indicated by red circles. In (**d**), the colours indicate water molecule diffusion occurring in the right/left (red), anterior/posterior (green) and superior/inferior (blue) directions. Also shown is an example of voxel-based analysis of FA data obtained using FSL’s Tract-Based Spatial Statistics (TBSS), specifically (**e**) a WM skeleton overlaid on an Montréal Neurological Institute (MNI) standard brain, and (**f**) voxels on this skeleton which are significantly different between two populations under study, represented in orange.

DT-MRI measures the random motion of water molecules *in vivo*. In free water, diffusion is isotropic, that is the movement of water molecules is equal in all directions. Conversely, in the brain’s WM the motion of water molecules is restricted by axonal membranes and myelin, so diffusion is not equal in all directions, and is therefore anisotropic [16]. This random motion can be represented mathematically by the diffusion tensor **D** [17]. This 3-by-3 symmetric matrix, which is measured in each imaging voxel within the brain, can be decomposed into three eigenvalues and three eigenvectors which indicate the magnitude and directionality of diffusion in three orthogonal directions [18]. In WM, since water molecules diffuse preferentially along the principal fibre direction, the eigenvector corresponding to the largest eigenvalue is taken to represent the fibre direction. This information can be used in a technique called tractography (see below) to examine connectivity between different brain areas [19]. Furthermore, the three eigenvalues can be employed to generate biomarkers of WM structure, the commonest of which are the mean diffusivity (MD), which measures the magnitude of water molecule diffusion, and fractional anisotropy (FA), which measures its directionality coherence (Figure 1a–d); FA takes values from 0, which indicates completely isotropic diffusion, to 1, which indicates completely anisotropic diffusion [18].

In healthy WM, MD typically varies from approximately 600 to 1200 × 10^−6^ mm^2^/s, while FA takes values in the range 0.5 to 0.8. However, if WM is compromised, MD will be higher and FA lower indicating reduced restriction of water molecule diffusion by cellular structures. These biomarkers are quantitative and can be compared across groups and individuals to look at differences in WM structure. For more detailed overviews of DT-MRI concepts, see Le Bihan *et al.*, (2001) [20] and Jones (2008) [21].

Currently, there are no standard conventions regarding the analysis of DT-MRI data, rather there are numerous techniques in existence that can be used either exclusively or in combination with each other to measure MD and FA, depending on the population under study and the hypothesis to be tested. The subsequent section will describe some of the different approaches to DT-MRI analysis including Region of Interest (ROI) analysis, voxel-based methods such as Tract-Based Spatial Statistics (TBSS) [22] and quantitative tractography techniques, in conjunction with a description of their relative advantages and disadvantages.

### 2.2. Region of Interest Analysis

ROI analysis usually involves an expert observer hand-drawing features, such as focal lesions, on structural MRI data and transferring them to co-registered DT-MRI data to measure diffusion biomarkers (Figure 1a–c). Although this is most often performed manually, automated methods of ROI placement have been suggested to improve objectivity; see Snook *et al.*, (2007) [23] for more details. ROIs are used to restrict the areas of the brain in which measurements are made, thereby reducing the need for statistical corrections for multiple-comparisons across the whole brain. ROI analysis is also useful for measuring the relative size of anatomical features between subjects or groups. Once the ROIs are selected MD and FA can be compared in these regions between subjects or time-points. A considerable advantage of the ROI technique is that it allows the investigation of differences in native space within individual brains, so details are not lost in the registration process to a standard space template, as occurs in voxel-based methods (see below). However, ROI analysis does require a strong hypothesis in order to select suitable regions for comparison, along with control regions. ROI analysis is also extremely labour intensive as manual placing, visual inspection and editing is required for each of the selected regions to ensure the same structure is measured in each person.

### 2.3. Voxel-Based Analysis

Voxel-based analysis can be used to investigate WM differences on a voxel-by-voxel basis across the brain. TBSS [22], which was specifically designed to analyse DT-MRI data by reducing misregistration artefacts between subjects and partial volume averaging of GM and cerebrospinal fluid signal with WM [25], is currently the closest to a standard analysis method for DT-MRI. In TBSS, the FA map for each participant is registered to a standard space template, a skeleton created indicating the centre of each WM tract, and then statistical analysis performed on a voxel-by-voxel basis to test for group differences (see Figure 1e,f). Due to the requirement that corrections are performed for multiple comparisons across the many hundreds of voxels that constitute the WM skeleton in each subject, TBSS is a relatively conservative analysis technique, typically requiring upwards of 20 to 30 subjects to provide robust significant differences between groups. Furthermore, the requirement that each subject’s FA data is registered to a standard space template may cause loss of small individual differences that are present in the native space data. Nevertheless, it is entirely automated and can be used to look at whole brain WM differences in a hypothesis-free manner.

### 2.4. Tractography Techniques

**Figure 2:**
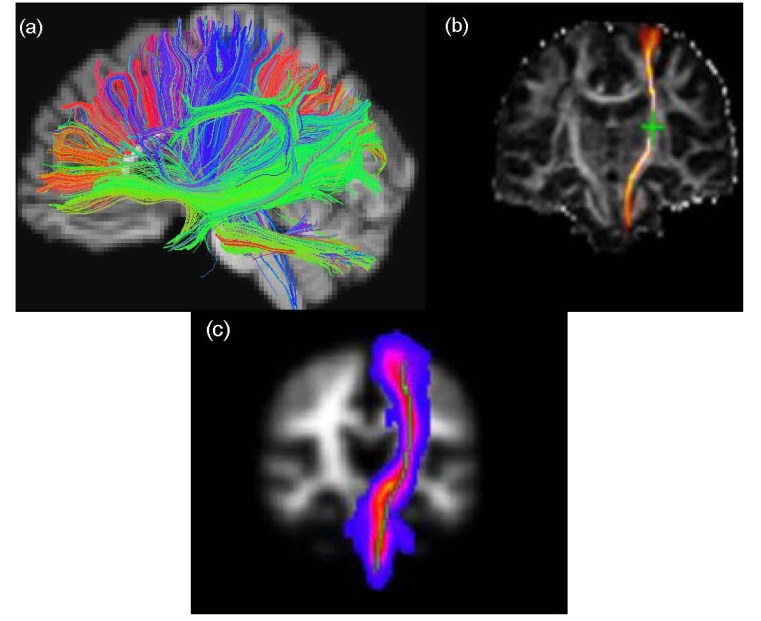
Examples of the visualization of WM tractography data. The figure shows (**a**) whole brain WM overlaid on a high-resolution T_1_-weighted volume scan produced by TrackViz [31], (**b**) left corticospinal tracts of the same participant (CST) generated using FSL’s BedpostX/ProbTrackX algorithm and (**c**) maximum intensity projection of a standard space group map of left CST obtained from 90 participants aged over 65 years using TractoR [32,33].

Tractography aims to reconstruct major WM tracts in 3D by piecing together voxel-based estimates of the underlying continuous fibre orientation field starting from an initial seed point [19] (Figure 2). Since tractography output can be highly sensitive to the choice of initial seed point placement, a number of different approaches have been suggested to allow the same tract to be identified in different subjects across a population (e.g., Conturo *et al.*, 1999 [26]; Clayden *et al.*, 2007 [27]). As discussed below, there are two common types of fibre tracking algorithm that can be employed for tractography analysis, namely deterministic and probabilistic, which differ in the way they deal with multiple fibres within a single voxel.

#### 2.4.1. Deterministic Tractography

Deterministic tractography assumes that the eigenvector associated with the largest eigenvalue of **D** is parallel to the fibre direction within each voxel, so that following this principal eigenvector direction will allow a single WM tract to be reconstructed in 3D space [19]. Tracts are terminated based on anisotropy and curvature thresholds, though at present there are no standard conventions to determine these thresholds. Furthermore, although deterministic methods can produce anatomically realistic tracts, they generally suffer from the problem that only a single fibre population within each voxel can be accurately modelled from **D**. Since at the resolution of DT-MRI (typically 1.5 to 3 mm in each voxel dimension), approximately one third of voxels contain more than one fibre population and these fibres often cross or “kiss”, this can be a significant drawback in the accurate representation of the underlying brain structure [28].

#### 2.4.2. Probabilistic Tractography

A different approach to generating WM tracts from diffusion MRI data is provided by probabilistic algorithms. These methods typically replace a single fibre direction with sampling from a distribution of orientations generated from a less restrictive model of the diffusion signal, e.g., a combination of a single perfectly isotropic (ball) and multiple anisotropic (stick) Gaussian compartments [28]. This provides an estimate of the uncertainty of connection and the ability to track through regions of low diffusion anisotropy or voxels containing several fibre populations with differing orientations. In these probabilistic methods, a measure of connectivity from a specified voxel to the initiating seed-point is provided by the percentage of pathways launched from the latter that reach the former. For these reasons, probabilistic tractography is generally considered to provide a more accurate method for determining fibre direction and tract reconstruction than deterministic tractography [29,30].

### 2.5. Applications to Musicians

The analysis techniques outlined above have been used to study a range of WM fibre tracts in musicians and non-musicians, such as the CC, sensory tracts such as the corona radiata, motor tracts such as the CST, the internal capsule in the basal ganglia and the AF, connecting temporal and frontal areas. To the best of our knowledge, 12 studies to date have investigated WM differences in musicians using DT-MRI, with the majority of these published within the last 5 years. As discussed above, methodological differences, for example deterministic or probabilistic tractography, should be taken into account when comparing the outcomes of these studies. Whilst some trends appear to be emerging from the range of findings across studies, there are also a number of inconsistencies and apparently conflicting results, which will be discussed next (see Table 1).

## 3. Using DT-MRI to Investigate the Effects of Musical Training on White Matter Architecture

Two of the most common study designs used to assess the effects of musical training are cross-sectional and longitudinal. In the former, two matched cohorts (e.g., age, gender and handedness) except for the variable of interest, musical training, are compared, whereas the latter compares changes within the same group of participants before and after a period of musical training. To the best of our knowledge, to date, only cross-sectional designs have been used to investigate the potential effects of musical training on WM architecture using DT-MRI.

### 3.1. Cross-Sectional Studies

Schmithorst and Wilke pioneered the use of DT-MRI to investigate WM differences between musicians who had continuous musical training during childhood and adolescence (duration ≥ 10 years) (*n* = 5), and non-musicians (*n* = 6) [34]. The authors used voxel-based analysis and reported that musicians displayed significantly greater FA in the genu of the CC, which connects the prefrontal cortices, but significantly lower FA in the corona radiata and internal capsule, through which both efferent and afferent motor and sensory fibres pass. A more recent study by Steele and colleagues [35] used a combination of TBSS and ROI analysis to compare 18 early trained (ET) musicians who commenced their training before the age of seven, with 18 late trained (LT) musicians who commenced their training after the age of seven, and 17 non-musicians. Results revealed significantly greater FA in ET musicians compared with both other groups in the posterior midbody of the CC, which connects the sensorimotor regions between hemispheres, and in the anterior portion of the isthmus, which joins the body and splenium of the CC. The authors also found that probabilistic tractography confirmed the finding of increased FA in the isthmus of ET musicians compared with the other two groups. Thus, the findings of Steele and colleagues [35] complement and extend the earlier DT-MRI findings by Schmithorst and Wilke [34] and the structural MRI studies by Schlaug and colleagues and Hyde *et al.*, [11,13,14,15], thereby providing support for the hypothesis that musical training can induce changes in the CC, possibly during a sensitive period of development. Such differences in the corpus callosum may reflect the highly skilled bimanual motor coordination and auditory skills that musicians require in order to play their instrument. It should be noted though, that the corpus callosum is subdivided differently in different studies (e.g., in half or into seven segments) and indeed that the specific regions of the CC reported to be different in musicians compared with non-musicians is not consistent between these studies, requiring further clarification in future research.

**Table 1:** Overview of studies and findings.

Reference	No. of Participants	Analysis Method	Key Findings
Schmithorst & Wilke 2002 [34]	5 Musicians 6 Non-Musicians	Voxel-Based	Significantly greater FA in the genu of the corpus callosum, but significantly lower FA in the corona radiata and internal capsule in musicians compared with non-musicians
Bengtsson *et al.*, 2005 [36]	8 Pianists 8 Non-Pianists	Voxel-Based	Significantly greater FA in the right posterior limb of the internal capsule in musicians compared with non-musicians. FA in several brain regions was positively correlated with mean total number of hours practice time in childhood, adolescence and adulthood.
Han *et al.*, 2009 [37]	18 Pianists 18 Non-Musicians	Voxel-Based	Significantly greater FA in the right posterior limb of the internal capsule in musicians compared with non-musicians. No significant correlation between either the age of training onset or total number of years training and FA.
Halwani *et al.*, 2009 [38]	11 Instrumentalists 11 Singers 11 non-musicians	ROI & Probabilistic Tractography	Tract volume of the arcuate fasciculus was greatest in singers, then instrumentalists and then non-musicians. FA in singers was significantly lower at the midpoint of the longitudinal portion of the left dorsal arcuate fasciculus compared with instrumentalists and non-musicians.
Imfeld *et al.*, 2009 [39]	13 Early Trained (ET) musicians 13 Late Trained (LT) Musicians 13 Non-Musicians	Deterministic Tractography, ROI & Voxel-Based	Significantly lower FA values in the CST of musicians compared with non-musicians. Significantly higher MD in both the left and right CST in ET musicians compared with LT musicians. No significant differences between absolute pitch (AP) musicians and non-AP musicians. No correlation between FA in the bilateral CST and age of training onset. MD in the CST was negatively correlated with age of training onset.
Oechslin *et al.*, 2010 [40]	13 AP Musicians 13 Non-AP Musicians 13 Non-Musicians	Deterministic Tractography & ROI	Correlation between AP ability and FA in the superior longitudinal fasciculus. AP demonstrated a greater-left-than-right asymmetry of FA in the superior longitudinal fasciculus.
Loui *et al.*, 2011 [41]	12 AP Musicians 12 non-AP Musicians	Deterministic Tractography & ROI	Higher volume and fibre number in tracts connecting the posterior superior temporal gyrus to the middle temporal gyrus in AP compared with non-AP musicians. Correlations between performance accuracy on a pitch-naming test, designed to test perfect pitch skills, and fibre volume connecting the left superior temporal gyrus and left middle temporal gyrus.
Abdul-Kareem *et al.*, 2011 [42]	10 Musicians 10 Non-Musicians	ROI & Deterministic Tractography	Significantly greater right middle cerebellar peduncle volume, right superior cerebellar peduncle volume and number of streamlines in right superior cerebellar peduncles in musicians compared with non-musicians. No correlation between age of training onset and WM volume differences or number of streamlines.
Dohn *et al.*, 2013 [43]	17 AP Musicians 18 Non-AP Musicians	TBSS	Significantly greater FA in a single WM cluster within the path of the inferior fronto-occipital fasciculus, uncinate fasciculus and the inferior longitudinal fasciculus in AP compared with non-AP musicians. AP ability associated with a rightward FA asymmetry.
Steele *et al.*, 2013 [35]	18 ET Musicians 18 LT Musicians 17 Non-Musicians	TBSS, ROI & Probabilistic Tractography	Significantly greater FA in the posterior midbody of the corpus callosum, and in the anterior portion of the isthmus in ET musicians compared with both LT musicians and non-musicians. Age of training onset correlated with FA in the posterior midbody of the corpus callosum.
Rüber *et al.*, 2013 [44]	10 Keyboard Players 10 String Players (Violin and Cello) 10 Non-musicians	Probabilistic Tractography Voxel-wise analysis within the tracts	Significantly greater FA in PT in right hemisphere of string players and keyboard players compared with non-musicians. Significantly greater FA in the PT in the left hemisphere of pianists. FA values in left and right PT and aMF significantly correlated with maximal tapping speed of the contralateral index finger.
Engel *et al.*, 2014 [45]	18 Non-Musicians	TBSS	FA values in the bilateral CST and right superior longitudinal fasciculus were correlated with learning speeds of piano melodies with the right hand.

MD = Mean Diffusivity; FA = Fractional Anisotropy; ROI = Region of Interest; TBSS = Tract-based spatial statistics; ET = Early Trained; LT = Late Trained; CST = Corticospinal Tract; PT= Pyramidal Tracts; aMF=Alternate Motor Fibres; AP = Absolute Pitch.

The finding of lower FA in internal capsule as reported in the initial study by Schmithorst and Wilke is challenged by two later studies, the first by Bengtsson and colleagues [36] and the second by Han and colleagues [37], both of which also used voxel-based analysis techniques. Bengtsson and colleagues reported significantly greater FA in the right posterior limb of the internal capsule in pianists (*n* = 8) compared with non-musicians (*n* = 8) [36], a finding that was later replicated by Han and colleagues using a larger sample size of pianists (*n* = 18) and non-musicians (*n* = 21) [37]. However, a study by Imfeld and colleagues [39] comparing musicians (*n* = 26) with non-musicians (*n* = 13), which used deterministic tractography, ROI analysis and voxel-based analysis, reported significantly lower FA values in the CST, which pass through the posterior limb of the internal capsule, carrying impulses mainly from the motor cortex to the contralateral side of the body. Furthermore, Imfeld and colleagues [39] reported that the ET musicians (*n* = 13) had a significantly higher MD in both the left and right CST compared with the LT musicians (*n* = 13). Low FA and high MD values are both generally associated with ageing or diseased WM, so interpretation of this finding in musicians is difficult, although the possibility of motor skills becoming highly automated has been offered as one possible explanation [34]. These conflicting reports of increased or decreased FA within the internal capsule of musicians, may be driven by differing analysis techniques and/or different types and numbers of musicians recruited, but at the moment there is a not a clear pattern emerging from which to draw strong conclusions.

The important question of whether specialist abilities are likely to be linked with increased or decreased FA is specifically addressed by four studies that have investigated WM architecture in a sub-group of musicians who possess perfect or absolute pitch (AP), that is, the ability to identify accurately the correct name of any pitch without a given reference point. In one study, deterministic tractography and ROI analysis have revealed correlations between error rates on an absolute pitch performance test and FA in three clusters in the left superior longitudinal fasciculus (*i.e.*, high performance on the AP-test was associated with low mean FA values), which connects the frontal, parietal, temporal and occipital lobes [40]. Meanwhile, Loui *et al.*, [41] used deterministic tractography and ROI analysis and demonstrated positive correlations between performance accuracy on a pitch-naming test, designed to test perfect pitch skills, and fibre volume connecting the left superior and left middle temporal gyrus [41]. Additionally, it has been suggested that AP may be associated with a higher FA in the left compared with the right SLF, *i.e.*, a greater-left-than-right asymmetry [40], yet this finding is disputed by a later study which used TBSS and reported a rightward asymmetry [43]. As the authors of the latter study state, TBSS is a relatively conservative technique, which may account for the differences reported in earlier studies not being replicated here [43]. However, TBSS analysis did indicate that AP musicians had significantly higher FA in a single WM cluster within the path of the inferior fronto-occipital fasciculus, UF and the ILF compared with non-AP musicians [43]. Contrastingly, Imfeld and colleagues [39] reported no significant differences between AP musicians (*n* = 13) and non-AP musicians (*n* = 13), so subsequently collapsed the two groups into a single group of musicians for additional analysis. Collectively, these studies of AP musicians provide some evidence that specialised skills may be associated with increased FA in association fibres, although, like the reports of increased and decreased FA in musicians in the internal capsule, the findings are mixed. It may of course be possible that different brain regions respond differently to musical training, or indeed that different kinds of musical training may have different plasticity effects. In addition the aetiology of perfect pitch, *i.e.*, whether it is genetically determined or due to early exposure to music, is highly debated [41], so it is not easy to infer whether any WM differences observed between AP and non-AP musicians are necessarily due to musical training or due to prior, innate differences in brain structure.

WM plasticity has been further examined by investigating structural differences between different types of musicians, which is interpreted as lending stronger support to the idea of training-related rather than innate differences. For example, Halwani *et al.*, [38] compared singers (*n* = 11), instrumentalists (*n* = 11) and non-musicians (*n* = 11) using hand-drawn ROI and probabilistic tractography and found that the tract volume of the left AF was significantly greatest in singers, compared with both instrumentalists and non-musicians. Interestingly though, ROI analysis along the AF revealed that FA in singers was significantly lower at the midpoint of the longitudinal portion of the left dorsal AF compared with instrumentalists and non-musicians, interpreted as possibly reflecting an increase in microstructural complexity. More recent evidence of inter-musician differences is provided by a study by Rüber and colleagues [44], which used probabilistic tractography and voxel-wise analysis within the tracts, to investigate between-group WM motor tract differences in keyboard players (*n* = 10), string players (*n* = 10), and non-musicians (*n* = 10). The right hemisphere of both string players and keyboard players was reported to show significantly greater FA in the pyramidal tracts (PT) and alternate motor fibres (aMF), compared with non-musicians, whereas in the left hemisphere only pianists displayed significantly greater FA values in these regions, interpreted as reflecting the different fine motor skill demands for pianists (bilateral) and string players (left hand). In addition, FA values in both the left and right PT and aMF were significantly correlated with maximal tapping speed of the contralateral index finger, indicating that FA differences in these tracts might occur in correlation with musical skill acquisition. The studies described above provide some evidence that different types of musical training, e.g., string, keyboard or vocal, may induce different WM plasticity, due to the specific skills developed. In addition, it appears that musical training may influence WM tracts related to motor functions. However, pre-existing differences resulting in a predisposition for fast tapping speeds and/or a preferred kind of musical instrument cannot be excluded.

Another key question with regard to whether differences observed between musicians and non-musicians are genetically determined or training-induced, is whether or not neuroplastic changes occur in correlation with the amount of musical practice and/or the age of onset of musical training. Gaser and Schlaug [4] found correlations between practice intensity associated with musicianship status (professional, amateur or non-musician) and GM volume in primary motor cortex, premotor cortex and cerebellum. However, evidence of a correlation between WM architecture and practice intensity appears to be mixed. In their study involving 18 pianists, Han and colleagues [37] found no significant within-group correlations between either the total number of years training or the age of training onset and FA in any brain regions. Similarly, Imfeld *et al.*, [39] found no correlation between FA in bilateral CST and age of training onset in their study of 26 professional musicians comprised of 13 AP musicians and 13 non-AP musicians. However, the authors did report that MD in the CST was negatively correlated with age of training onset, and that this effect was due to a subgroup of ET musicians [39]. A study conducted by Abdul-Kareem and colleagues found increased right middle and superior cerebellar peduncle WM volume and increased number of fibres in the right superior cerebellar peduncle in professional musicians (*n* = 10) compared with non-musicians (*n* = 10), but no significant correlation between age of training onset or duration of musical training and either WM volume or number of fibres in the middle and superior cerebellar peduncles [42]. In contrast to the above, Steele and colleagues [35] reported a significant correlation between FA in the posterior midbody of the CC and age of training onset. Furthermore Bengtsson *et al.*, [36] reported positive correlations between hours of practice time and FA in different brain regions for different age periods, suggesting that FA changes due to musical training may be related to WM tract maturation trajectories. This somewhat conflicting evidence as to whether or not FA is correlated with practice duration or age of training onset means it is not possible at this time to draw firm conclusions regarding specific brain regions. It may be possible that FA is only correlated with age of training onset if training commences during a sensitive learning period, possibly before the age of seven, reflecting a non-linear correlation between FA and age of training onset. Alternatively, differences in WM tracts have been observed between different types of musicians [32,44], so it is possible the mixed evidence may be due to inter-musician differences. Differences in the total number of years of training and performance level attained may also affect results, as well as differences in analysis techniques, ROIs and statistical power. Thus, further longitudinal studies similar to the structural MRI study by Schlaug and colleagues [13,14,15] are still needed in order to determine whether WM changes occur in correlation with musical skill acquisition and whether pre-existing WM differences can predict musical skills.

### 3.2. Experimental Musical Training Paradigms

The authors of the majority of the cross-sectional studies outlined above conclude that WM differences observed between musicians and non-musicians are induced by musical training, yet pre-existing differences in WM cannot be excluded while evidence as to whether these differences are correlated with training duration or age of training onset is mixed. Although no longitudinal studies have to date been performed with DT-MRI to investigate differences in WM architecture pre and post-musical training, Engel and colleagues investigated the speed at which non-musicians (*n* = 18) learnt to play short piano melodies in two different training conditions: either a visuo-motor condition in which the participants received no auditory information or an auditory-motor condition where the participants’ view of their hands was obstructed [45]. DT-MRI scans were acquired after 3 days of training, totalling approximately 2–5 h, in one of the two training conditions. Data were analysed using TBSS and FA values in bilateral CST and right SLF were reported to be correlated with the learning speeds of piano melodies with the right hand in the audio-motor condition, *i.e.*, higher FA values were correlated with faster learning speeds. No significant differences in FA value were reported between participants who completed the two different training conditions, although if the effect size were small it potentially would not have been registered by TBSS. The most straightforward interpretation of this result is that pre-existing WM differences reflect a predisposition for learning musical skills, thus somewhat undermining the hypothesis that structural differences found between musicians and non-musicians occur predominantly as a result of musical training.

### 3.3. DT-MRI and Motor Skills

When considering the variety of DT-MRI findings regarding the effects of musical training, and resulting issues, it is perhaps useful to view these in the context of other DT-MRI studies on the effects of motor skill acquisition. In recent years, a growing number of studies have used DT-MRI to assess training-related brain change after cognitive interventions [46,47] or motor skill learning (for reviews and critical commentary see Thomas and Baker [48] or Chang [49]). Here, somewhat inconsistent findings in cross-sectional studies of long-term motor training are also reported, for example the lower FA values found bilaterally under the premotor cortex of female ballet dancers [50], but increased FA in the CST of gymnasts [51]. Longitudinal designs examining more short-term training have shown increased FA in the WM tracts underlying the intraparietal sulcus after practising juggling five days a week for six weeks [52], and increased FA in the internal capsule, corona radiata and CC after 9 sessions of complex bilateral visuomotor training [53], but performance-related decrease in prefrontal FA after six weeks of weekly balance board training [54]. The latter finding was interpreted by the authors to be due to learning-related increase in cell density or axonal/dendritic arborisation hindering water diffusion, which could perhaps be related to an early stage of learning. Stage of learning does seem likely to have an impact in such research, for example, reduced MD in hippocampus and parahippocampus have been reported after training periods as short as two hours on a car-racing computer game [55]. Of course, ballet, gymnastics, juggling and balance board training are all highly bilateral motor activities engaging a broad range of musculature as well as visual processing, and thus it is difficult in such studies to specify a highly precise skill-related movement and related neural ROI for investigation and hypothesis. A more precise motor task that has been used to investigate multimodal processing is visuomotor tracking with a joystick [56], which after seven consecutive days training was shown to increase FA in the area underneath the primary motor cortex of the hand used to practise, thus lending support to the finding previously reported in gymnasts [51]. Similarly, a relatively simple 4-week training paradigm aimed at increasing unilateral leg strength was found to lead to a decrease in MD in the contralateral CST (although this was the only tract under investigation) [57]. Collectively then, the motor training studies in this field further underscore the complexity of DT-MRI research findings and their interpretation, underlying the need for future studies in this area. Furthermore, as most studies of motor training have not included questions about a critical developmental window, this currently remains an open question in the field, and perhaps one where musical training studies might make a particular contribution.

## 4. Discussion

Whilst the majority of published studies suggest that there is evidence that WM plasticity can be induced by musical training, the nature and extent of such plasticity remains under discussion, and there is also some evidence that pre-existing WM differences may be predictors of musical learning ability. It thus appears likely that the frequently reported structural brain differences observed between adult musicians and non-musicians are a consequence of both genetic and environmental factors, still to be precisely determined.

In addition, it should be acknowledged that many of the papers reviewed here lacked the descriptive methodological clarity required for replication, for example with regard to the precise data analysis technique employed, or with regard to participant information such as instrument played and age of commencing training. Furthermore, a number of non-significant findings, or findings that did not survive correction for multiple comparisons, are presented alongside statistically significant findings. As noted in Baker and Thomas [48], unclear reporting and statistical issues also appear in the majority of papers on longitudinal studies of brain change related to motor learning, and as such this problem is not specific to music studies, but a broader issue in research focusing on brain plasticity and skill acquisition.

Nevertheless, to date, differences in the CC have been regularly reported in musicians compared with non-musicians in studies using DT-MRI [34,35] and studies employing image segmentation of structural T1-weighted scans [11,13,14,15]. Several studies investigating CC structures report differences in regions where the fibres primarily connect motor related brain regions [11,13,14], which likely reflects the bimanual coordination and related inter-hemispheric connections required for playing most musical instruments. In addition, differences in WM architecture are regularly reported between ET musicians, who commenced their training before the age of seven and LT musicians, who commenced their training after the age of seven [11,35,36,37,39]. Han and colleagues [37] noted that the statistical power of their findings was lower than that of Bengtsson *et al.*, [36] despite having a much larger group of participants, which they suggested may be due to their participants commencing their training at a mean age of 12.2 years, compared with the participants in the earlier study who began their training at a mean age of 5.8 years. Also, as mentioned above, Steele *et al.*, [35] reported significantly higher FA values in the posterior midbody of the corpus callosum in ET musicians compared with LT musicians, despite the participants having completed on average the same number of years training. Critical learning periods for musical training have been further investigated in terms of behavioural performance, including rhythm synchronisation [58,59] and cognitive abilities [60]. It appears that even when matched for total number of years’ experience, years of formal training and current number of hours practice, ET musicians still perform better than LT musicians on rhythmic synchronisation tasks. Watanabe *et al.* suggest this reflects a sensitive period during which intense motor training can result in sustained performance advantages later in life [59]. Furthermore, it has been suggested that the relationship between age of training onset and performance on a rhythm synchronisation task is non-linear and that a critical motor learning period could extend up until around the age of nine reflecting underlying GM and WM maturation trajectories [58]. In addition, a recent paper by Bailey and colleagues [61] reported increased GM in the right ventral premotor cortex of ET musicians compared with LT musicians and non-musicians. Moreover GM in this region was correlated with performance on a rhythmic synchronisation task [61]. These studies appear highly consistent with the findings of Schlaug and colleagues [11,13,14] and the above-mentioned DT-MRI studies [31,32,33] providing support for idea of a critical period, during which the brain has the most potential to undergo neuroplastic changes. However, this critical learning period seems to be somewhat flexible in terms of specific age cut-off, and WM structural plasticity is of course not restricted to this period. No studies to date have specifically investigated musical training-induced structural plasticity in the healthy adult brain, but evidence from longitudinal studies of motor learning e.g., juggling, suggests that WM plasticity is possible in adulthood [52]. Furthermore, it was reported that FA values in the WM tracts underlying right posterior interparietal sulcus remained elevated 4-weeks after the juggling training ceased, perhaps suggesting that the FA change was sustained after training had ceased [52], while this was not found to be the case for GM changes [48,62].

Reports of FA differences in the internal capsule between musicians and non-musicians are conflicting, with reports of both increased [36,37] and decreased FA in musicians compared with non-musicians [34]. Support for the latter finding is provided by the report of lower FA values in the CSTs as they pass through the posterior limb of the internal capsule in musicians compared with non-musicians [39], although it should be noted that the CSTs are not the only WM tracts to pass through the posterior limb of the internal capsule. FA increases reported in the internal capsule may be due to FA increases in the corticobulbar tracts for example, since Rüber and colleagues reported increased FA in PT, which is comprised of both the CST and corticobulbar tracts [44]. Evidence from AP musicians lends some support to the idea that that specialised skills are associated with increased FA values. Since instrumentalists are required to have highly developed fine motor skills, it might be expected that connectivity should be increased and FA in motor tracts such as the CST would be greater in musicians compared with non-musicians. Indeed, increased FA in the CST of gymnasts [51], another population group required to have fine motor control, provides supporting evidence that highly developed motor skills may be associated with increased FA values in motor tracts, although this finding was contradicted in ballet dancers [50]. A recent study by James and colleagues [63] of GM density across 59 expert, amateur and non-musicians adds to this discussion, as it provides evidence that performance related increases can be observed in brain regions involved in higher order cognitive processing, whilst decreases are observed in sensorimotor areas. It seems possible that something similar may be observed in terms of FA in WM tracts, such that FA in the CC increases with expertise whilst FA in the CST decreases with expertise. However, this explanation does not account for reports of both increased and decreased FA within the same brain region, for example the internal capsule. Reports of FA differences between musicians and non-musicians in other brain regions including the AF and cerebellar peduncles are from single studies, with relatively small numbers of participants where the findings have not been replicated to date, so further evidence is required before these findings can be accurately interpreted.

Evidence as to whether age of training onset or practice duration are correlated with FA values in different brain structures, including the CC, cerebellar peduncles and CST is also inconsistent [35,36,37,39,44]. Possible reasons for the contrary results can be grouped into two broad categories: recruitment of participants and differences arising from the various DT-MRI acquisition and analysis techniques, which will now be discussed in turn.

### 4.1. Effects of Participant Recruitment

In general the number of participants included in DT-MRI studies is small, which presents problems with statistical power. Some of the DT-MRI analysis techniques, particularly TBSS, are only effective with a relatively large sample size (typically 20 or more), so increasing the sample size would provide a solution to issues arising from statistical power. Whilst some of the currently available studies have used a homogenous sample of musicians, e.g., pianists, other studies have used different types of instrumentalists or not specified the types of musician at all, although WM differences between instrumentalists and singers have already been reported [38], as have differences between AP and non-AP musicians [40,41,43]. The only study to date to have specifically investigated WM differences between different types of instrumentalists reported differences in WM architecture between string players and keyboard players [44]. In addition, a study of GM reported structural brain differences in the hand-motor area occur between string players and keyboard players [3]. Collectively these studies suggest that the choice of musicians recruited (e.g., instrument played and AP ability) should be carefully considered in future research. Since there is reasonably consistent evidence of WM differences between ET and LT musicians, these groups should also perhaps be treated as distinct samples. In addition, the type of musical training (e.g., Suzuki) musicians have undertaken should be perhaps taken into consideration as teaching methods vary and often prioritise different skills, such as auditory learning *versus* notation-based learning.

Evidently, longitudinal studies are going to be essential in order to fully investigate questions about pre-existing neural differences, structural changes occurring in correlation with skill acquisition and the effects of age of training onset, practice intensity and training duration. Randomised longitudinal studies will be especially important, since there are other pre-existing factors (such as socio-economic status and academic achievement) that predict whether children will choose to start and continue with music training [64].

### 4.2. Effects of Analysis Methods

Developing our knowledge of WM plasticity relies on effective measurement and analysis techniques. DT-MRI offers unique insights into the brain’s connectivity, but variability in acquisition and analysis techniques, and lack of standard methodologies, complicates both interpretation and replication of results. DT-MRI requires high quality MRI data as free as possible from random subject motion, eddy current induced distortions and susceptibility artefacts [65], and is ideally acquired with whole brain coverage, isotropic voxels and 30 or more diffusion encoding directions [66]. Such data, which typically takes between 10 and 20 min to acquire, permits probabilistic tractography and gives the greatest chance of identifying WM differences between populations. More advanced acquisition schemes, such as diffusion spectrum MRI [67], which make no assumption about the form of the diffusion signal, can also be used in combination with tractography to resolve multiple crossing fibres in the brain [68]. However, such methods require substantial amounts of diffusion MRI data to be collected, resulting in a much longer scan time, which renders it unfeasible in many situations, although the availability of ultra-high field (7+ Tesla) scanners may change this situation in the future [69]. Moreover, as it was recently demonstrated that MD as measured by DT-MRI requires a smaller sample size than some of the more elaborate diffusion metrics to detect group effects, it may be preferable over other metrics that account for more inter-subject variability, but need much larger sample sizes [70].

A further degree of detail can be provided by combining structural and diffusion MRI to investigate whole-brain structural connectivity [71]. In “connectomics”, the aim is to generate a complete map of neural connections by describing the brain as a large structural network made up of neural connections (WM tracts) and neural units (GM regions). Cortico-cortical connections can be identified by measuring the number of WM tracts generated by tractography between all pairs of cortical regions produced by parcellating the cortex from high-resolution structural MRI scans. Computational network analysis can then generate metrics such as node degree, node strength, sparsity, network efficiency and centrality which may potentially serve as useful biomarkers for studying structural connectivity in normal development and neurological disorders [29]. As yet, however, no study has applied this technique to examine the effects of music training on brain connectivity, although Jancke *et al.*, [72] have used the associated technique of “local connectedness” derived completely from structural MRI to show that AP musicians (*n* = 13) have increased local connectivity in brain regions known to be involved in higher-order auditory processing compared with non-AP musicians (*n* = 16) and non-musicians (*n* = 12).

DT-MRI analysis techniques are still under development and there are currently no standard conventions as to how this data is best analysed in all situations, although TBSS is probably closest to a standard. However, all methods have strengths and drawbacks. ROI analysis is powerful in situations where placement is obvious, such as in focal lesions. Where WM structure appears normal, ROI placement can be subjective and prone to error. Voxel-based methods, such as TBSS, are automated and therefore more objective. However, as they perform statistical comparisons over many hundreds of voxels, they can be overly conservative and require more subjects than ROI methods to find significant differences. They also typically warp each subject’s data into a common space, which may reduce or remove the very individual differences the study was designed to measure. Tractography has the potential to map the 3D structure of WM pathways *in vivo*, a capability not shared by any other technique. Validation studies with animal models show good correspondence between tractography results [73], although care should still be taken to interpret the results in light of known anatomy [21]. Tractography can also be used to automatically measure MD and FA biomarkers in tracts of interest, a potentially more objective approach than provided by ROI analysis. However, seed points should be placed with care to ensure the same structure is identified in each subject to allow meaningful comparisons [26]. Choice of tracking algorithm is also important with probabilistic approaches generally preferred to deterministic, due to their ability to resolve regions of crossing fibres. With this in mind, studies of musicians that have used probabilistic tractography [35,38,44] could, arguably, be given more weight than those that have used deterministic tractography [39,40,41,42] (although of course other design factors should also be considered important). These different analysis techniques, which potentially lead to inconsistent findings between studies, are important considerations when designing future experiments. Additionally, given the impact of these methodological choices, it is crucial to attain a reporting standard that always includes these analysis details.

## 5. Conclusions

The growing empirical evidence suggests that there are differences in WM architecture between musicians and non-musicians, and that pre-existing conditions as well as training-related effects can lead to WM differences, but the paucity in longitudinal studies and variation in methods currently preclude strong conclusions. The only DT-MRI study to date that investigated WM differences induced by musical training suggested that learning speeds showed the strongest correlation with differences in WM architecture [45]. However, the training duration in this study was only three days, so a longer intervention period, such as the 29 months received by the children who participated in the study by Schlaug and colleagues [13,14], may be required for any changes to occur. Nevertheless, shorter training periods have been reported to lead to plastic changes in non-musical motor learning and the length of training needed to have an effect on the brain change may also be dependent on age. Large-scale longitudinal studies are the only way to investigate whether pre-existing differences in WM are reflected in musical abilities, whether WM differences occur in correlation with musical skill acquisition and to investigate the duration and intensity of training required for any WM neuroplasticity to occur.

Diffusion methods have a unique ability to provide potential insights into WM connectivity *in vivo* that can be used to investigate structural changes in response to musical training in both cross-sectional and longitudinal studies. However, this potential is tempered by the lack of standard approaches to image acquisition and processing, which makes comparison of results from different studies challenging. At present, voxel-based analysis, such as TBSS, of high resolution, low artefact DT-MRI data from studies with large sample sizes is closest to this ideal, although quantitative tractography based on probabilistic tracking is also a candidate. To make further progress towards understanding how musical training affects WM structure, future studies should be well powered, employ longitudinal designs controlling for demographical variables that may also impact on WM development and use standard quantitative analysis procedures to ensure greater reliability and reproducibility of results.
